# Case report: A longitudinal study of an unusual rapidly progressive dementia case

**DOI:** 10.3389/fneur.2023.1151130

**Published:** 2023-04-06

**Authors:** Xiaoyan Liu, Ziqi Fan, Xuanyu Chen, Yanyan Zhang, Fangping He, Xiaohua Ma, Qing Ke

**Affiliations:** ^1^Department of Neurology, The First Affiliated Hospital, Zhejiang University School of Medicine, Hangzhou, China; ^2^Department of Neurology, The Second Division of Xinjiang Production and Construction Corps, Korla Hospital, Xinjiang, China

**Keywords:** autoimmune encephalopathy, IgLON5, immunotherapy, psychosis, rapidly progressive dementia

## Abstract

It is daunting to determine the etiology of rapidly progressive dementia (RPD), which includes metabolic, neoplastic, infectious, autoimmune, neurodegenerative and other conditions. Herein, we illustrate an unusual case of a patient primarily exhibiting RPD, overlapping sleep dysfunction, psychosis and abnormal movement, which was finally defined as anti-IgLON5 disease, a novel and rare autoimmune encephalopathy. Furthermore, we longitudinally described his cognitive and psychological performance in detail, and determined that early initiation of immunotherapy in this patient did not result in a good outcome. These data highlight anti-IgLON5 disease as a possible differential diagnosis in patients with RPD.

## Introduction

Rapidly progressive dementia (RPD) is a complicated condition, that originates from various types of etiologies, including vascular, infectious, toxic, metabolic, autoimmune, iatrogenic, neurodegenerative and other conditions (1). Hence, it is quite challenging to determine the etiology of RPD quickly. A novel neurological disease, anti-IgLON5, characterized by antibodies against the neuronal cell-adhesion protein IgLON5 has been gradually described since 2014 (2). This disease has been pathologically determined to involve tau inclusions; it is clinically characterized by chronic sleep disorder, accompanied by gait instability, bulbar symptoms, and cognitive impairment (3). Nonetheless, the current understanding of this disease is incomplete, and very few cases have been reported worldwide (4). Herein, we report a case of a patient with anti-IgLON5 disease who exhibited prominent RPD. In particular, we longitudinally describe his cognitive and psychological performance as well as his clinical outcome in response to immunotherapy and symptomatic treatment.

## Case report

A 74-year-old, right-handed male was admitted to our hospital with jaw and bilateral hand movements for 3 months and RPD for 1 month, coupled with intermittent visual hallucinations and raving. Especially, he exhibited multiple involuntary movements (jaw tremor, motor tremor of hands), his hands always groped about, and would fell backward when he was walking or seated. Besides, he could not recognize his family or his location; sometimes he observed a snack moving around or a dead man in the room. He was sent to a local hospital and administered olanzapine 2.5 mg per night without marked improvement. Thus, he was transferred to our hospital. His past history was traced back to 5 years ago when he suffered from rectal carcinoma and received surgery and chemotherapy.

On admission, neurologic examination revealed prominent cognitive impairment and psychiatric symptoms. Specifically, he failed to complete the Mini-Mental State Examination (MMSE) and had a Neuropsychiatric Inventory (NPI) score of 50 (Table 1), which included hallucination, agitation, disinhibition, irritability, aberrant motor behavior and night-time behavior. The right dorsal interosseous muscle and thenar muscle were atrophic, the muscle strength of his four extremities was reduced but somewhat incompatibility, and the muscle tone was elevated; bilateral Babinski signs were negative, In addition, his hands groped about, and he fell back when sitting. The cranial nerves, sensory system and reflexes were intact.

**Table 1:** Results of longitudinal cognitive and psychiatric evaluations.

MMSE item	First evaluating score	Second evaluating score	Third evaluating score	Fourth evaluating score
Time frame	On admission (before immunotherapy)	On the 9th day of immunotherapy	On the 22nd day of immunotherapy	3 months after discharge
Orientation	Not completed	6/10	4/10	5/10
Registration		2/3	3/3	3/3
Attention and calculation		0/5	0/5	0/5
Recall		0/3	0/3	0/3
Language and praxis		4/9	5/9	4/9
Total MMSE score		12/30	12/30	12/30
NPI item				
Delusion	0	4^*^3, 3	0	0
Hallucination	4^*^3, 3	4^*^3, 3	0	0
Agitation	3^*^2, 3	4^*^3, 3	0	0
Depression/dysphoria	0	3^*^1, 1	3^*^2, 1	3^*^2, 1
Anxitey	0	4^*^2, 1	3^*^2, 1	3^*^2, 1
Euphoria	0	0	0	0
Apathy	0	0	0	0
Disinhibition	2^*^1, 2	0	0	0
Irritability	3^*^2, 3	4^*^3, 3	2^*^1, 2	2^*^1, 2
Aberrant motor behavior	4^*^3, 2	4^*^3, 2	4^*^2, 1	4^*^2, 1
Night-time behavior	4^*^3, 5	4^*^3, 5	3^*^2, 3	3^*^2, 3
Appetite/eating changes	0	0	0	0
Total NPI score	50	83	28	28
Total disruption score	18	21	8	8

The subscales of NPI were expressed in frequency ^*^ severity, disruption score.

His clinical features supported lesions located in the cerebral cortex and basal ganglia and a probable diagnosis of cerebellum and anterior horn. Considering his tumor history, lack of trauma or poisoning, extensive involvement of the nervous system and subacute onset, etiological diagnoses were narrowed to tumor/paraneoplastic syndrome, inflammation or infection. Thus, we created the following workup.

**Figure 1:**
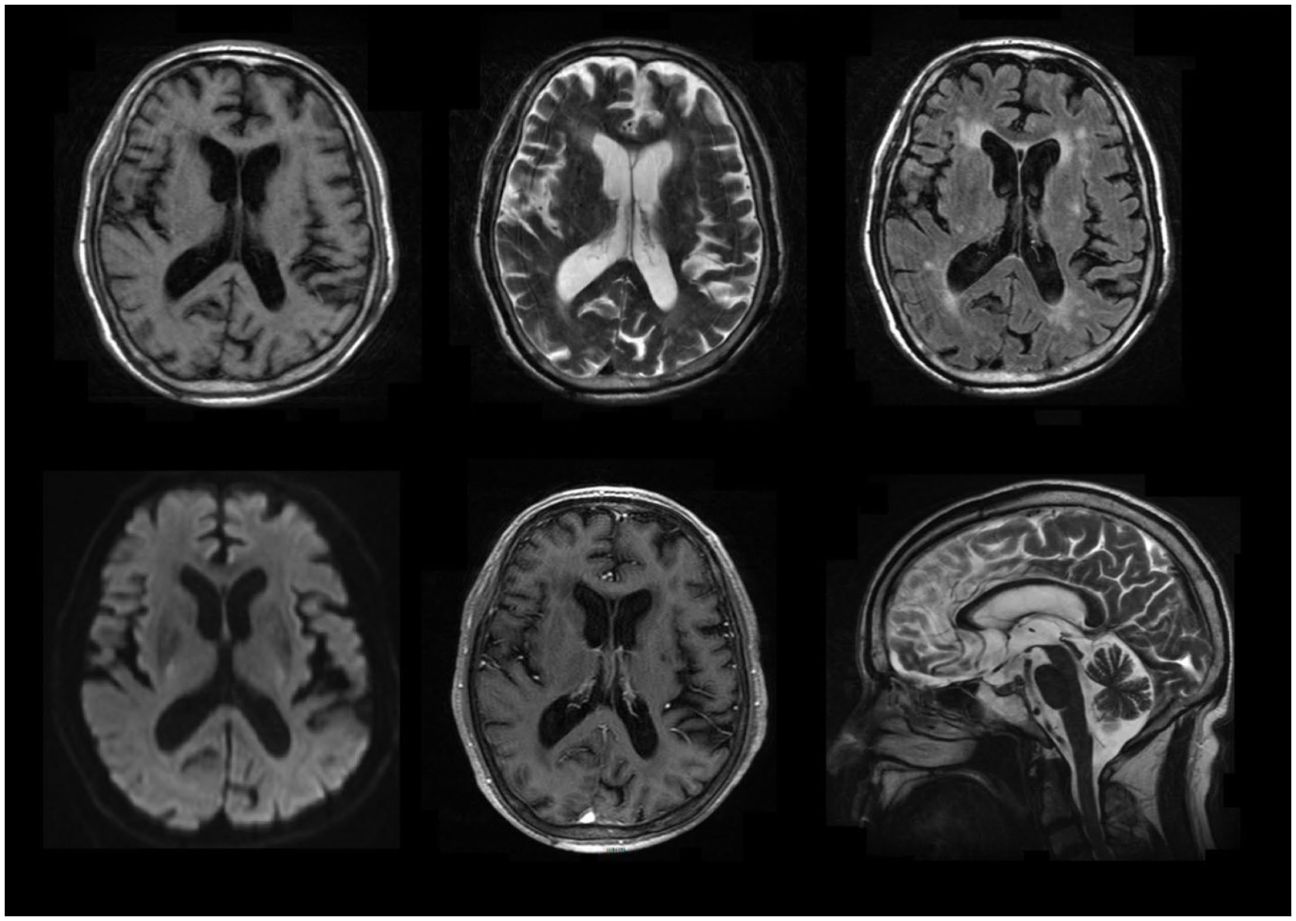
Selected brain MR images. It showed ischemia within the periventricular area and centrum semiovale but revealed no contrast-enhanced lesions.

**Figure 2:**
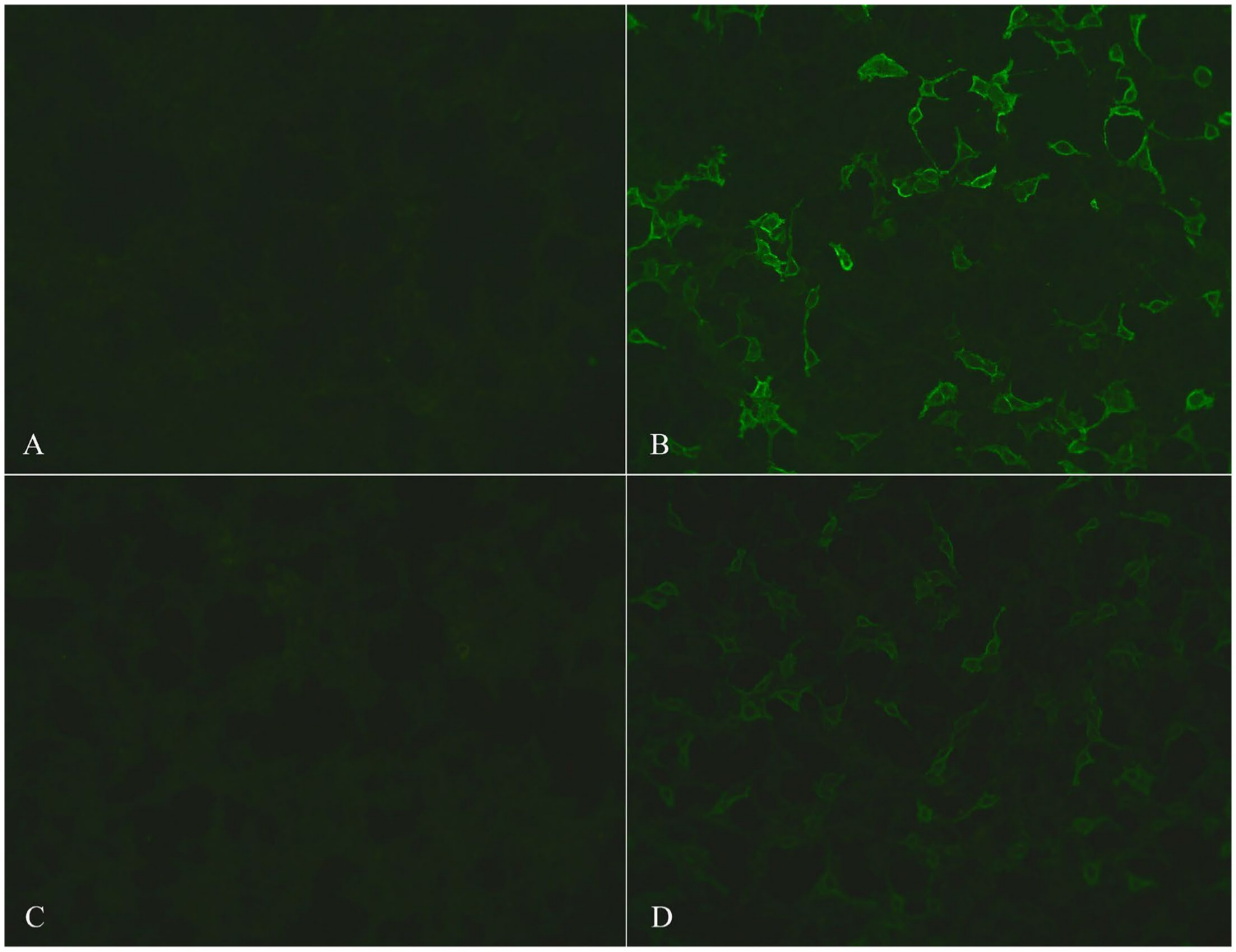
Photos of serum and CSF autoantibody screening. The photos of control-transfected cells in serum and CSF are shown in **(A)** and **(C)**, respectively; positive anti-IgLON5 antibodies in serum (++, 1:100) and CSF (++, 1:3.2) are shown in **(B)** and **(D)**, respectively.

MR imaging suggested ischemia within the periventricular area and centrum semiovale but revealed no contrast-enhanced lesions (Figure 1). Hematological screening was unremarkable, including a routine blood test, biochemistry, tumor marker, folate, vitamin B12, and thyroid function, as well as screenings for HIV, syphilis, and hepatitis B. A routine cerebrospinal fluid (CSF) test indicated normal pressure, leukocytosis (0 × 106/L), glucose and chlorine but mildly elevated protein levels (0.60 g/l, normal range 0.15–0.45 g/L). In serum and CSF autoantibody screening, anti-NMDA receptor, AMPA receptor 1 or 2, GABA-B receptor, LGI1, CASPR2 and DPPX6 IgG antibodies were negative, but anti-IgLON5 IgG antibody (serum: ++, 1:100; CSF: ++, 1:3.2) was positive, as shown in Figure 2. Human leukocyte antigen (HLA) typing did not reveal the presence of the HLA-DQB1*0501 or HLA-DRB1*1001 allele. A video electroencephalogram revealed increased slow activities without epileptic discharge. Ambulatory polysomnography (PSG) was conducted from 10 pm to 7 am and revealed extended stage N1 and N2 time, as well as shortened N3 and REM time; however, sleep efficiency was normal at 85.9%. During sleep time, he experienced 6 obstructive sleep apneic events, with an apnea hypopnea index of 6.7/h, and all 6 apnea events occurred during NREM sleep. The longest and mean apnea times were 42 s and 28 s, respectively. Hypopnea occurred 34 times during NREM sleep and once during REM; the longest and mean hypopnea times were 172 s and 89.4 s, respectively. In addition, snoring occurred 438 times and accounted for 5% of his sleep time. His minimum SPO2 (64%) was recorded during NREM. However, no stridor was detected. Periodic leg movements occurred 50 and 51 times during NREM and wakefulness, respectively, with a mean power and duration of 17 dB and 1.7 s, respectively. Taken together, his PSG data suggested an aberrant sleep mode, characterized by disorganized sleep cycles, obstructive sleep apneic events, excessive snoring and increasing periodic leg movements. Nevertheless, PET-CT and electromyography data were unavailable because of his noncompliance. Therefore, he was confirmed to have anti-IgLON5 disease.

**Figure 3:**
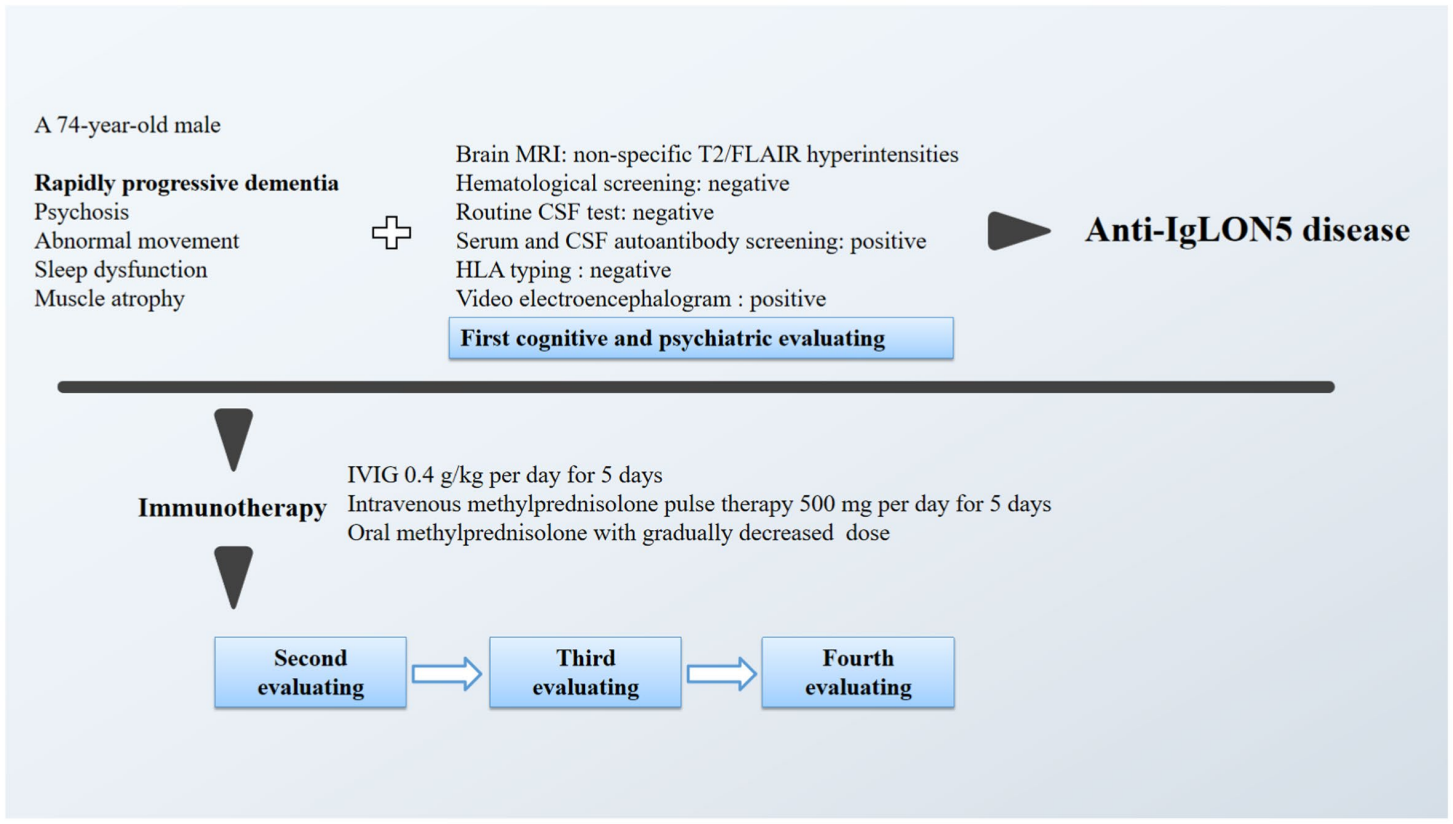
A schematic diagram of the clinical characterization and management of this male.

After hospitalization, he continued to take olanzapine 2.5 to 5 mg per night and was administered pramipexole and compound levodopa. When his diagnosis was confirmed, he was also treated with immunotherapy, including intravenous immunoglobulin (IVIG) 0.4 g/kg per day for 5 days, followed by intravenous methylprednisolone pulse therapy 500 mg per day for 5 days. Then, methylprednisolone was decreased to 80 mg per day, and mycophenolate mofetil 500 mg was administered twice a day. A follow-up evaluation was performed, as shown in Table 1. On the 9th day of immunotherapy, he was cooperative for MMSE evaluation, showing a total score of 12/30 and an improvement in orientation, immediate recall and naming and repetition function. However, his NPI score did not improve and indicated more severe delusion, agitation and irritability. Specifically, he accused his daughter and wife of attempting to kill him and intermittently shouted at and kicked them. On the 22nd day of immunotherapy, his MMSE score remained the same, but his NPI score decreased to 28; he mainly exhibited depression, anxiety, irritability, aberrant motor behavior and night-time behavior without hallucination, delusion or agitation. He was discharged home on the 26th day of immunotherapy and continued to take olanzapine, pramipexole, compound levodopa, mycophenolate mofetil and prednisolone orally 60 mg per day, with a decrease of 10 mg per month. At this time, his muscle atrophy and weakness remained at the same level, and abnormal movement had disappeared. Follow-up contacts at 3 and 6 months after discharge did not show further improvement, and follow-up contact at 12 months showed a final outcome of death due to cerebral hemorrhage. A schematic diagram of the clinical characterization and management of this male is shown in Figure 3.

Assays for CSF and serum autoantibody IgG were performed using cell-based indirect immune-fluorescence tests employing BIOCHIPs (EUROIMMUN AG, Luebeck, Germany) at EUROIMMUN Diagnostic Laboratory, China. Written informed consent for publication was obtained from the patient’s legally authorized representative.

## Discussion

Anti-IgLON5 disease is a rare and novel neurological disease. Fortunately, we adopted an up-to-date autoantibody screening method including a new item “anti-IgLON5 antibody” in this patient, especially when his sleep disruptions were unapparent. This finding is a humbling reminder that our clinicians should utilize new screening techniques and be aware of easily overlooked clinical signs.

Here, we report a Chinese male with anti-IgLON5 disease who exhibited prominent RPD, overlapping sleep dysfunction, psychosis, abnormal movement, muscle atrophy and weakness. To our knowledge, RPD usually occurs over months, weeks or days. Given the established etiological categories of RPD, prion diseases are the leading causative factor; apart from neurodegenerative factors, autoimmune conditions are the second most common causative factor for nonprion RPD. In the literature, VGKC, Yo, Hu, Ma, CV2, GAD65 and other antibodies mediate autoimmune diseases (5). However, the current understanding of anti-IgLON5 disease is incomplete. To date, a few studies have examined this disease with RPD. Considering the rapid progression (< 3 months for memory deficits, which is considered rapid cognitive decline), a study reported this presentation in 3 patients with anti-IgLON5 disease and suggested that RPD was not a frequent clinical manifestation of this disease. Notably, patients in that study did not develop psychiatric symptoms (6). In addition, a recent study reported a female patient with anti-IgLON5 disease who presented with rapidly progressive cognitive decline, and she showed atypical inflammatory lesions on MRI, as well as HLA-DRB1*1001 and HLA-DQB1*0501 association (7). In contrast to other cases, our patient mostly exhibited rapid, extensive cognitive impairment that impacted orientation, execution, memory and language function. For example, he could state his name but could not identify his wife and daughter, state where he was or when it was, calculate 100 minus 7, or repeat what we said. Those issues occurred within 1 month; thus, these features were consistent with a diagnosis of RPD. Simultaneously, this man initially exhibited psychotic positive symptoms, including hallucination, agitation, disinhibition and irritability. In addition, the characteristics of PSG were evidenced by his sleep disruption. However, this male patient did not show HLA-DRB1*1001 and HLA-DQB1*0501 associations.

Furthermore, we longitudinally described his cognitive and psychological performance in detail and explored his response to immunotherapy and symptomatic treatment. With respect to immunotherapy options, IVIG combined with methylprednisolone and mycophenolate mofetil was administered to this patient. Notably, this therapy was associated with mild improvement in cognition at the initial stage, especially in the domains of orientation, registration and language function; as a consequence, he could somewhat communicate with others on the 9^th^ day of immunotherapy. However, this performance did not further improve afterward, and prominent impairment of attention, calculation and recall function persisted. Moreover, his psychotic positive symptoms resolved and were replaced by psychotic negative symptoms, such as depression and anxiety. A longitudinal follow-up of his clinical course at 12 months revealed a poor outcome. This case indicated by this case, that immunotherapy combined with symptomatic treatment was partially effective for anti-IgLON5 disease at an early stage.

However, the exact mechanism of anti-IgLON5 antibody in this clinical manifestation, disease course and improved response remains unclear. Previous studies have indicated an intriguing association between tauopathy and anti-IgLON5 disease. Neuropathological features suggested noninflammatory pathology with tau hyperphosphorylation and accumulation in several brain regions, preferentially involving the hypothalamus, brainstem tegmentum and upper spinal cord (8), and appeared to support a chronic disease course. A possible explanation may lie in the membrane stabilization of IgLON5. The rapid disruption of IgLON5 may have induced corresponding tau hyperphosphorylation and accumulation; in turn, antibody-mediated effects repaired this disruption (9). HLA association might be a reason for disease recovery (8). Thus, we assume that the improved response may be associated with the opportunity to initiate this therapy; earlier treatment is better, especially before the formation of tauopathy.

## Conclusion

We longitudinally described a patient with anti-IgLON5 disease, particularly his presentation of RPD and the curative effect. Our findings support the hypothesis that anti-IgLON5 antibody-mediated effects and HLA association modulate the disease course and its improved response, further promoting our understanding of the mechanisms underlying cognitive and psychological recovery. In addition, anti-IgLON5 disease should be considered a possible differential diagnosis in patients with RPD.

## Data availability statement

The original contributions presented in the study are included in the article/supplementary material, further inquiries can be directed to the corresponding authors.

## Ethics statement

Written informed consent was obtained from the patient’s legally authorized representative for the publication of this case report.

## Author contributions

XL, ZF, and QK participated in the clinical treatment and writing of the study. XC, YZ, FH, and XM participated in the sample collection and analyses of the results. XL and QK gave the pivotal answers and guidance to the manuscript revision. All authors contributed to the article and approved the submitted version.

## Funding

This work was supported by the National Natural Science Foundation of China (81801056).

## Conflict of interest

The authors declare that the research was conducted in the absence of any commercial or financial relationships that could be construed as a potential conflict of interest.

## Publisher’s note

All claims expressed in this article are solely those of the authors and do not necessarily represent those of their affiliated organizations, or those of the publisher, the editors and the reviewers. Any product that may be evaluated in this article, or claim that may be made by its manufacturer, is not guaranteed or endorsed by the publisher.

## References

[1] Geschwind MD Haman A Miller BL. Rapidly progressive dementia. Neurol Clin. (2007) 25:783–807, vii. doi: 10.1016/j.ncl.2007.04.001, PMID: 17659190 PMC2706263

[2] Sabater L Gaig C Gelpi E Bataller L Lewerenz J Torres-Vega E. A novel non-rapid-eye movement and rapid-eye-movement parasomnia with sleep breathing disorder associated with antibodies to IgLON5: a case series, characterisation of the antigen, and post-mortem study. Lancet Neurol. (2014) 13:575–86. doi: 10.1016/S1474-4422(14)70051-1, PMID: 24703753 PMC4104022

[3] Simabukuro MM Sabater L Adoni T Cury RG Haddad MS Moreira CH. Sleep disorder, chorea, and dementia associated with IgLON5 antibodies. Neurol Neuroimmunol Neuroinflamm. (2015) 2:e136. doi: 10.1212/NXI.0000000000000136, PMID: 26236762 PMC4516399

[4] Graus F Santamaria J. Understanding anti-IgLON5 disease. Neurol Neuroimmunol Neuroinflamm. (2017) 4:e393. doi: 10.1212/NXI.0000000000000393, PMID: 28852690 PMC5570673

[5] Geschwind MD Shu H Haman A Sejvar JJ Miller BL. Rapidly progressive dementia. Ann Neurol. (2008) 64:97–108. doi: 10.1002/ana.21430, PMID: 18668637 PMC2647859

[6] Escudero D Guasp M Ariño H Gaig C Martínez-Hernández E Dalmau J. Antibody-associated CNS syndromes without signs of inflammation in the elderly. Neurology. (2017) 89:1471–5. doi: 10.1212/WNL.0000000000004541, PMID: 28878050 PMC5631166

[7] Montagna M Amir R De Volder I Lammens M Huyskens J Willekens B. IgLON5-associated encephalitis with atypical brain magnetic resonance imaging and cerebrospinal fluid changes. Front Neurol. (2018) 9:329. doi: 10.3389/fneur.2018.00329, PMID: 29867738 PMC5966542

[8] Gelpi E Höftberger R Graus F Ling H Holton JL Dawson T. Neuropathological criteria of anti-IgLON5-related tauopathy. Acta Neuropathol. (2016) 132:531–43. doi: 10.1007/s00401-016-1591-8, PMID: 27358064 PMC5023728

[9] Hashimoto T Yamada M Maekawa S Nakashima T Miyata S. IgLON cell adhesion molecule Kilon is a crucial modulator for synapse number in hippocampal neurons. Brain Res. (2008) 1224:1–11. doi: 10.1016/j.brainres.2008.05.069, PMID: 18602091

